# Altered Error Processing following Vascular Thalamic Damage: Evidence from an Antisaccade Task

**DOI:** 10.1371/journal.pone.0021517

**Published:** 2011-06-23

**Authors:** Jutta Peterburs, Giulio Pergola, Benno Koch, Michael Schwarz, Klaus-Peter Hoffmann, Irene Daum, Christian Bellebaum

**Affiliations:** 1 Institute of Cognitive Neuroscience, Department of Neuropsychology, Faculty of Psychology, Ruhr University Bochum, Bochum, Germany; 2 Department of Neuroscience and Faculty of Biology and Biotechnology, Ruhr University Bochum, Bochum, Germany; 3 Department of Neurology, Klinikum Dortmund, Dortmund, Germany; University of Alberta, Canada

## Abstract

Event-related potentials (ERP) research has identified a negative deflection within about 100 to 150 ms after an erroneous response – the error-related negativity (ERN) - as a correlate of awareness-independent error processing. The short latency suggests an internal error monitoring system acting rapidly based on central information such as an efference copy signal. Studies on monkeys and humans have identified the thalamus as an important relay station for efference copy signals of ongoing saccades. The present study investigated error processing on an antisaccade task with ERPs in six patients with focal vascular damage to the thalamus and 28 control subjects. ERN amplitudes were significantly reduced in the patients, with the strongest ERN attenuation being observed in two patients with right mediodorsal and ventrolateral and bilateral ventrolateral damage, respectively. Although the number of errors was significantly higher in the thalamic lesion patients, the degree of ERN attenuation did not correlate with the error rate in the patients. The present data underline the role of the thalamus for the online monitoring of saccadic eye movements, albeit not providing unequivocal evidence in favour of an exclusive role of a particular thalamic site being involved in performance monitoring. By relaying saccade-related efference copy signals, the thalamus appears to enable fast error processing. Furthermore early error processing based on internal information may contribute to error awareness which was reduced in the patients.

## Introduction

Performance monitoring and flexible behavioural control are necessary in order to adapt and to optimize behaviour in accordance with changing environmental demands. The neural underpinnings of error processing, i.e. the detection and correction of performance errors, involve a network of midbrain, basal ganglia (BG) and frontal cortical structures, with a prominent role of the anterior cingulate cortex (ACC) [1]–[4]. Electroencephalographic (EEG) research has identified the error negativity (Ne) [5] or error-related negativity (ERN) [6] as a correlate of error processing. The ERN is a negative event-related potential (ERP) with a frontocentral distribution peaking within approximately 100 to 150 ms after an erroneous response, and it is unaffected by conscious awareness of having made an error [5], [7]–[9].

Source localization analyses have shown that the ERN is generated in the ACC [10], [11]. Functional imaging results corroborate the importance of the ACC for error processing [4], [12]. Moreover, a pronounced reduction of the ERN was shown in a patient with a rare focal lesion of the rostral-to-middorsal ACC [13]. Reports of ERN attenuation in patients suffering from Parkinson's disease [3], Huntington's disease [14] and lesions to the basal ganglia [15] or the orbitofrontal cortex [16] indicate a crucial role of fronto-striato-thalamo-cortical feedback circuits for error processing and performance monitoring.

The short latency of the ERN with an onset around the start of the actual execution of an erroneous response, i.e. when the first electromyographic activity is observed [6], suggests that it is based on central information rather than peripheral feedback which is rapidly fed into an internal error monitoring system [17]. An “efference copy" of the motor command for a response may enable such a monitoring system to build a representation of the actual response prior to receiving sensory or proprioceptive feedback, and thus to compare it to a representation of the appropriate response [6], [18], [19]. Since an ERN-like negative deflection has also been observed on correct trials - the correct-response negativity (CRN) - [18], [19], the ERN has been suggested to reflect a process of response conflict evaluation [20] rather than a mere comparison process [18]. In accordance with this notion, the ERN has been shown to be larger under conditions of high response conflict, e.g. when opposing response options are similar, e.g. in the case of movements of the left versus right hand compared to movements of the left hand versus right foot [21]. On the other hand, findings of very similar ERPs for correct and error responses under conditions of performance uncertainty, i.e. when the available data for response evaluation is limited with regard to perceptual properties or attentional resources [22], clearly favour the error detection account. In any case, efference copy information may be highly relevant for the error monitoring system, as it can provide accurate information about the precise nature of the planned or ongoing response, which is necessary for determining potential mismatches between the representations of the appropriate and the actually performed response.

Typically, the ERN is observed in response time tasks in which subjects have to perform simple button presses in response to visual stimuli under speed instructions. The antisaccade task requiring subjects to suppress a reflexive eye movement towards a peripheral onset cue and to perform a fast saccade in the opposite direction has also been used to investigate error processing [7], [8], [23], [24]. Erroneous prosaccades reliably elicit an ERN [7], [8]. It has been suggested that most of these direction errors remain unrecognized because of parallel programming of a reflexive prosaccade and the antisaccade. The antisaccade command is generated in the frontal eye field (FEF) and projected to the deep layers of the superior colliculus (SC) and the premotor reticular formations of the brainstem [25]–[27]. A bottom-up command for a prosaccade, which is automatically processed by the deep layers of the SC and forwarded to the saccade-generating neurons in the brainstem, may be faster and bypass cortical control [24].

For a saccade to be performed, activity of the ocular motoneurons is rapidly increased in a pulse-step-like fashion [28]. At the same time, an efference copy of the motor command is also generated in order to enable monitoring of self-movement [29]. Electrophysiological research in primates [30]–[32] has identified a pathway from the SC to the FEF through the mediodorsal nucleus of the thalamus (MD) relaying efference copy information about ongoing saccades. Studies on patients with thalamic lesions strongly suggest that the processing of efference copy signals for the internal monitoring of saccades relies on the functional integrity of the thalamus [33]–[37]. In humans, deficits in processing efference copy information comparable to those described in monkeys with reversible MD lesions were observed after ventrolateral (VL) thalamic lesions [34], although MD may also play a role [33], [34], [37].

Previous work in humans suggested that efference copy signals are processed rapidly following a saccadic eye movement. ERP components associated with efference copy processing in the context of transsaccadic updating of visual space have been observed within 100 ms after a saccade, and this signal has been shown to be altered in thalamic lesion patients [38], [39]. The time course of saccade-related efference copy processing is thus compatible with the assumption that the ERN is based on such an internally generated signal. The finding of thalamic contributions to efference copy-based saccade monitoring therefore strongly suggests that the thalamus contributes to the processing of erroneous saccadic eye movements. The present study investigated error processing in patients with focal vascular damage to the thalamus by means of an antisaccade task. EEG was recorded to assess brain potentials in response to erroneous prosaccades and correct antisaccades. Consistent with findings of impaired processing of saccade-related efference copy signals following lesions of the thalamus [31], [32] and in accordance with the ERN error detection account [18], saccade-related ERPs were expected to distinguish less reliably between errors and correct performance in patients compared to controls.

## Methods

### Subjects

Six patients with focal thalamic damage due to ischemia in the putative territory of the paramedian artery (two men, four women) and twenty-eight neurologically healthy volunteers (12 men, 16 women) participated in the present study. Control subjects were recruited from a pool of volunteers at the Department of Neuropsychology at the Institute of Cognitive Neuroscience of the Ruhr University Bochum. Exclusion criteria for patients were current or past psychiatric disorders, current medication affecting the central nervous system, an IQ estimate of below 80, and past or current neurological problems apart from the thalamic lesion. The same criteria were applied for control subjects, with the exception that any type of neurological disorder led to exclusion from the study. All control subjects were right-handed as determined by the Edinburgh Handedness Inventory (EHI) [40]. Patient 2 was left-handed according to the EHI, but reported having been trained to use the right hand. All other patients were right-handed.

All subjects had normal or corrected-to-normal vision. All participants gave written informed consent prior to participation and received monetary reimbursement. The study conforms to the Declaration of Helsinki and received ethical clearance by the Ethics Board of the Medical Faculty of the Ruhr University Bochum, Germany.

**Figure 1:**
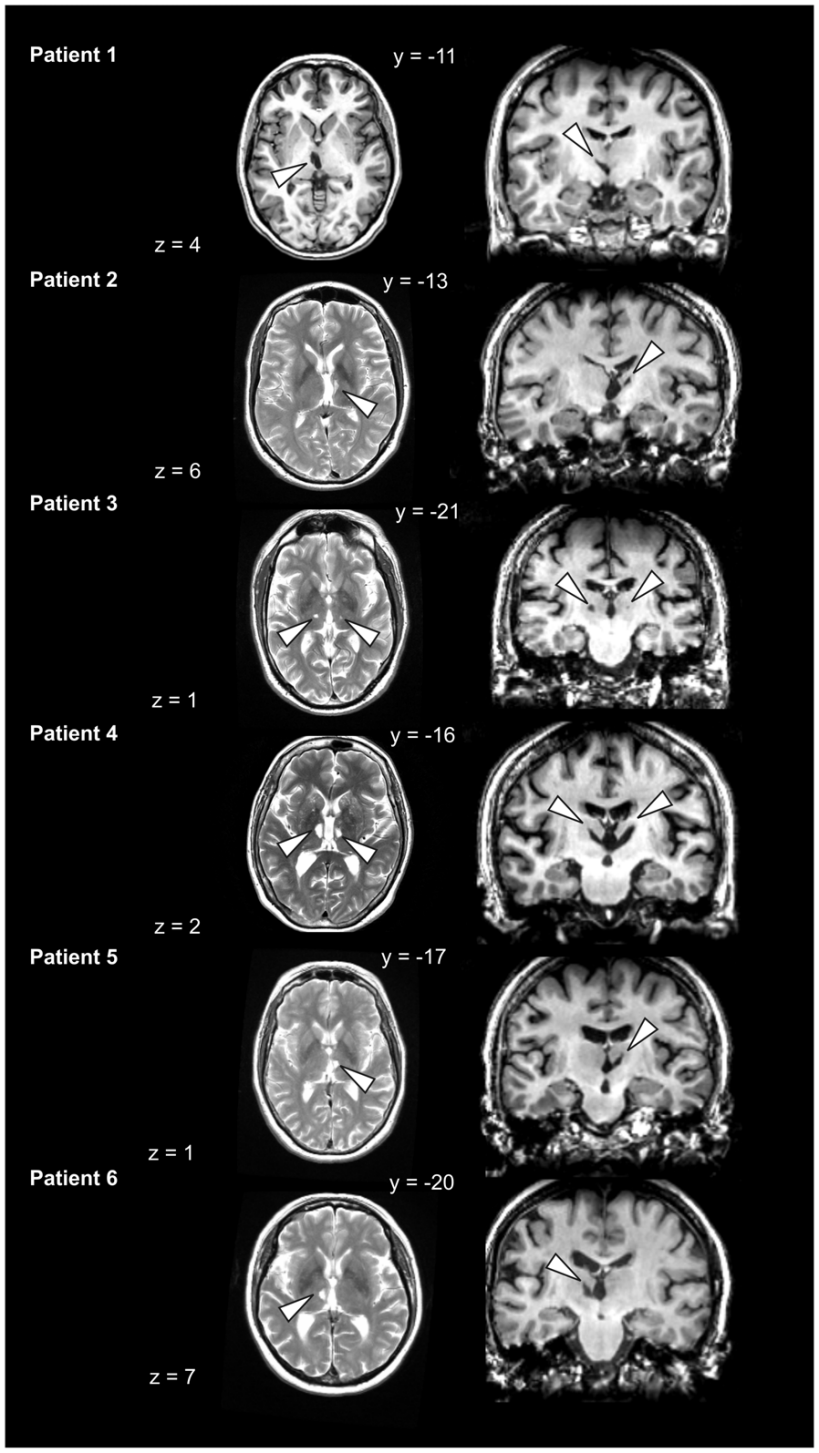
Transverse and coronal structural MR-images and lesion locations for all patients. T2-weighted transverse and T-1 weighted coronal MR images of lesion locations in individual patients (lesion locations are marked with white arrows). Note that for Patient 1 the transverse slice is also provided T1-weighted. MRI showed left-hemispheric damage to the thalamic region in Patient 1 and Patient 6, right-sided lesions in Patient 2 and 5 and bilateral damage in Patient 3 and Patient 4.

**Figure 2:**
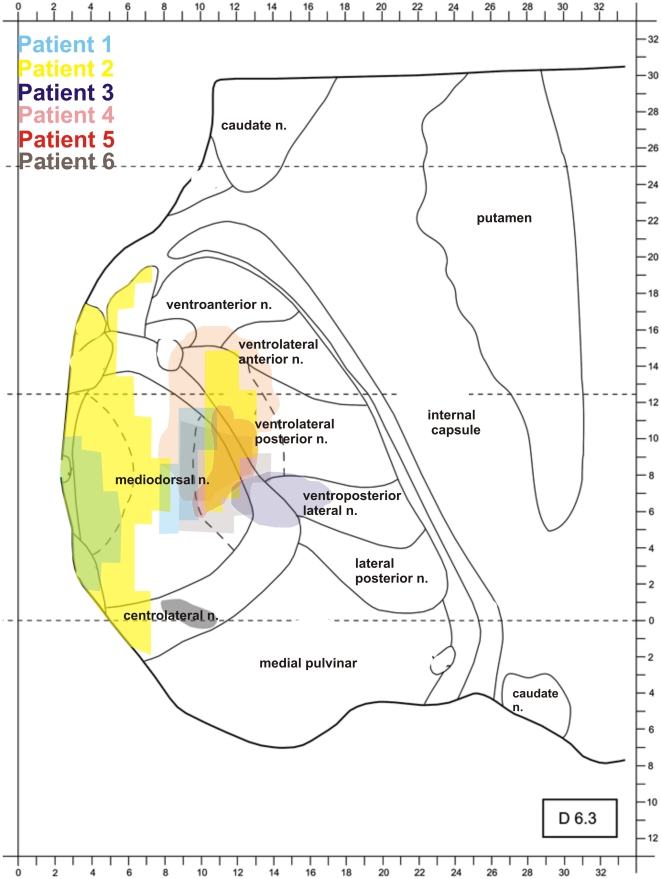
Overlay of the patients' lesion locations based on the stereotactic map of the thalamus. The map depicts stereotactic plane 6.3 and is oriented parallel to the intercommissural plane.

The patients, who will be referred to as Patients 1 to 6 in the following, were outpatients of the Klinikum Dortmund, Germany. As can be seen in Table 1, time since lesion varied considerably between patients. For diagnosis, lesions were documented with magnetic resonance imaging (MRI) using standard T1- and T2-weighted sequences for coronal and transverse sections (voxel size: 1 mm×5 mm×5 mm), respectively, upon initial admittance to the clinic. For research purposes, however, MR images with a higher resolution were obtained in a neurological follow-up examination, which took place within a period of ten months before participation in the study for Patients 1, 2, 4 and 5. For Patients 3 and 6, follow-up scans were performed two and nine months after participation, respectively. For the high resolution MRI, a standard axial T2-weighted sequence (voxel size: 0.5 mm×0.5 mm×5 mm) was used. Lesion location was specified by matching the individual patients' lesions onto corresponding schematic horizontal sections of the human thalamus provided by a stereotactic atlas [41]. Lesions were classified as primarily affecting MD, VL or both. Figure 1 depicts transverse and coronal high-resolution MR-images of the lesions for the six patients. For Patient 3, the initial MRI had shown damage to the left VL. Higher resolution MR during follow-up revealed that the lesion indeed primarily involved the left VL, but that there was also a much smaller lesion affecting VL on the right side. Similarly, Patient 2′s lesion initially had been classified to exclusively affect MD, but higher resolution MR during follow-up detected additional damage also in VL. Medially, thalamic shrinking seems to have occurred causing ventricular enlargement (see Figure 1), which is a common finding after paramedian strokes [42]. Figure 2 provides an overlay of the patients' lesion locations based on the stereotactic maps. Generally, patients did not report any residual symptoms majorly affecting their everyday lives. However, Patients 2 and 4 did report experiencing increased fatigue when dealing with demanding tasks and subtle (subjective) impairment of episodic memory.

**Table 1:** Age and IQ for patients and controls as groups, and for individual patients and their respective control groups as well as with time since lesion, affected nuclei and additional lesions for individual patients.

	Age	IQ	Time since	Affected nuclei	Additional lesion
			lesion (months)		
**Patient 1**	31	121.8	166	left MD,	None
*Controls (N = 10)*	34.6 (5.2)	113.5 (8.9)			
**Patient 2**	45	127.8	135	right MD, right VL	None
*Controls (N = 10)*	42.2 (5.3)	118.6 (10.3)			
**Patient 3**	52	122.5	136	left VL, right VL	None
*Controls (N = 10)*	51.1 (4.0)	120.8 (10.9)			
**Patient 4**	58	107.0	123	bilateral MD, left VL	Small cerebellar infarct
*Controls (N = 10)*	57.2 (5.9)	120.2 (9.9)			
**Patient 5**	62	109.8	39	right MD	None
*Controls (N = 10)*	58.3 (5.5)	119.5 (10.2)			
**Patient 6**	66	106	82	left MD	None
*Controls (N = 10)*	60.1 (6.1)	119.2 (10.2)			
**Controls mean (SD)**	47.0 (12.0)	117.7 (9.3)			
**Patients mean (SD)**	54.3 (12.5)	116.3 (8.6)			

MD  =  mediodorsal, VL = ventrolateral.

As will be outlined in more detail below, for the analysis of deficit patterns in single patients 10 control subjects were assigned to each individual patient based on the best matches with regard to age and IQ scores (see Table 1 and section 2.7. for details). IQ estimates were obtained using the “Picture Completion" and the “Similarities" subtests from the short German version of the Wechsler Adult Intelligence Scale [43]. On the group level, patients and controls did not differ in regard to age or IQ (both *p*>.164). None of the patients showed evidence of a scotoma, i.e. a lesion-induced visual field deficit which could have influenced performance on the antisaccade task, as determined by visual field screening [44].

### Experimental task

**Figure 3:**
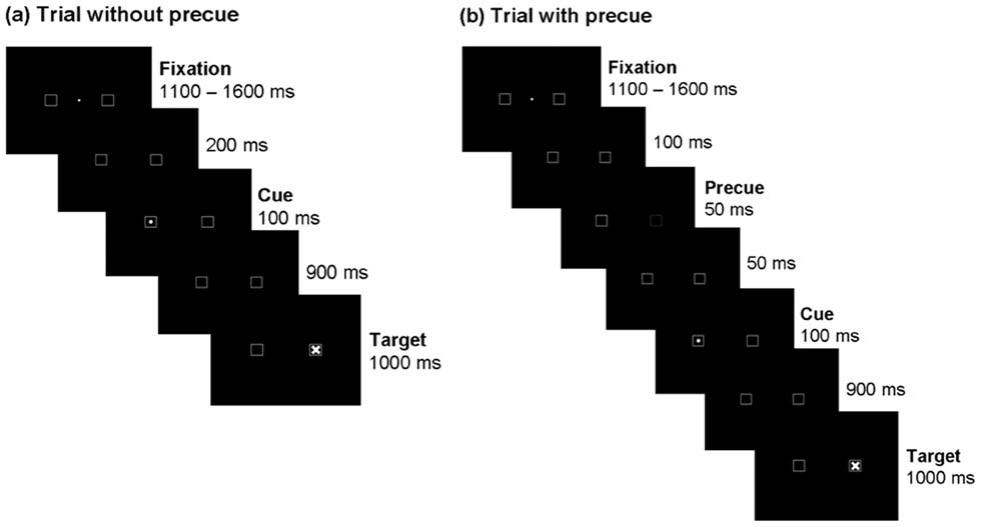
Antisaccade task. The antisaccade task: Upon onset of a peripheral stimulus (cue) in one of the two squares, subjects had to perform an antisaccade to the opposite square as fast and accurately as possible. The correct target location was marked at the end of each trial, and subjects were instructed to press a button if they had erroneously performed a prosaccade towards the cue. a) In half of the trials, no “precue" was shown, whereas in the other half of the trials b) a precue validly marked the target location briefly before the cue was presented. This procedure was introduced to increase error rates [45].

The time course of stimulus presentation in the antisaccade task is illustrated in Figure 3. Trials started with a white fixation dot (0.6°) located in the centre of the screen and two square frames (3° side length), the centres of which were located 8° to the left and right of the fixation dot. After a variable delay (1100–1600 ms), the fixation dot disappeared. There were two types of trials. In trials without precue, the peripheral squares remained unchanged for the next 200 ms. Subsequently a yellow dot (1° in diameter) was presented as cue stimulus for 100 ms unpredictably in the left or right square frame. In trials with precue, the colour of the square opposite to the cue location turned to red for the 50 ms duration between 100 and 50 ms before cue presentation, indicating the correct target location of the saccade (see Figure 3 for the sequence of events in both types of trials). Precues were introduced to increase error rates [45]. Participants were instructed to perform a single horizontal saccade as fast and accurately as possible to the square opposite to the position of the cue stimulus. 900 ms after cue offset, a white cross appeared, validly marking the target location for the saccade opposite to the cue. In accordance with previous studies using an antisaccade task [7], [8], error awareness was assessed by asking participants to report whenever they noticed performing an erroneous prosaccades. Participants were instructed to press the space bar if, *and only if*, they thought they had mistakenly moved their eyes towards the cue, i.e. made a pro- instead of an antisaccade. It was emphasized that button presses had to occur only while the target cross was visible in order to prevent hand movements during the cue-target interval.

The task comprised six blocks of 100 trials each. 20 practice trials were completed prior to the first block. Cue occurrence on the right or left side was balanced throughout the task. The stimuli were presented on a 17-inch computer screen with an LCD display. Subjects were seated at a viewing distance of 57 cm, a chin-rest stabilizing the position of the head. For the experiment, the room was darkened. Participants were free to take breaks between blocks. The entire session took about 60 minutes.

### Alertness and working memory

In order to determine if patients and control subjects differed with regard to cognitive abilities possibly relevant to the experimental task, reaction times on a tonic and phasic alertness task as well as performance on visual and verbal short-term and working memory tasks were assessed.

Alertness was investigated using an adaptation of the subtest ‘Alertness’ of a computerized German attention test battery [44]. In four blocks of 20 trials each subjects were required to respond to a visual target stimulus (X) by pressing a button as fast as possible (tonic alertness). At the beginning of each trial, a fixation dot was presented in the centre of the screen. After a variable delay of 2000 ms to 7000 ms the target stimulus was presented for up to 2000 ms. The target vanished as soon as the response button was pressed. In half of the trials, the stimulus was preceded by an auditory warning signal delivered 500 to 1500 ms prior to target onset. These trials assessed phasic alertness, i.e. the ability to increase attention in response to a warning stimulus. Reaction times were recorded separately for tonic and phasic alertness trials. If reaction time exceeded 2000 ms, participants were instructed to respond faster, and the trial was repeated at the end of the block. Verbal and visual short-term and working memory were assessed with the Digit Span and Block Span subtests from the Wechsler Memory Scale [46].

### Procedure

Participants were informed that the study investigated visuomotor integration. After signing the informed consent form, the electrodes were attached and the experiment was started. Alertness, visual field and short-term and working memory tasks were administered after the experimental task had been completed.

### Recording and analysis of saccade and EEG data

Movements of the participants' right eyes were captured using an iView X™ Hi-Speed video-based eye tracking system (SensoMotoric Instruments, Berlin, Germany) at a sampling rate of 500 Hz. Eye movement data were analyzed off-line using MATLAB (Mathworks, Natick, Massachusetts, USA). Saccades were identified by a velocity criterion (threshold, 40°/s) and a distance criterion (minimum saccade length 1.5°). Only those trials were further considered in the EEG analysis (see below), in which a horizontal saccade was performed from the fixation point towards either square frame, starting within the cue-target interval, i.e. within 1000 ms following cue onset. Saccadic reaction time (SRT) was determined as the time between cue onset and onset of the first saccade in the cue-target interval. Saccades in trials with SRTs shorter than 80 ms were considered anticipatory and discarded. Saccades were classified as correct antisaccades or errors (erroneous prosaccades). Contraversive saccades towards the target following a direction error were labelled ‘corrective saccades’. Correction time was determined as time between onset of the erroneous saccade and onset of the corrective saccade. Furthermore, the percentage of trials with erroneous prosaccades and corrective saccades as well as the percentage of recognized errors (aware errors) were determined. In order to investigate post-error slowing, SRTs on correct trials following errors and correct trials following correct trials were recorded [47].

Throughout the experiment, EEG was recorded from 30 scalp sites using a Brain Products BrainAmp Standard amplifier (Brain Products, Munich, Germany) and the appropriate software at a sampling rate of 500 Hz. Silver-silver chloride electrodes were mounted to an elastic cap according to the International 10–20 System (F7, F3, Fz, F4, F8, FT7, FC3, FCz, FC4, FT8, T7, C3, Cz, C4, T8, TP7, CP3, CPz, CP4, TP8, P7, P3, Pz, P4, P8, PO7, PO3, POz, PO4, PO8) and referenced to the linked mastoids, with impedances kept below 5 kΩ. EEG-data were analyzed off-line using BrainVision Analyzer 2 software (Brain Products, Munich, Germany) and MATLAB (Mathworks, Natick, Massachusetts, USA). Raw data were filtered with 1 Hz high-pass and 30 Hz low-pass filters. An independent component analysis (ICA) was performed on single-subject EEG data [48]. The ICA yields an unmixing matrix decomposing the multichannel scalp EEG into a sum of temporally independent and spatially fixed components, the number of these components matching the number of channels. Each component can be characterized by a unique time course and topographical distribution of activation. Each subject's 30 components were screened for components with a symmetric, frontally positive topography potentially reflecting blink artefacts or vertical eye movements. For each subject, one such component was identified and removed from the raw data by performing an ICA back transformation. In two control subjects, the back-transformed data still contained numerous blink artefacts upon subsequent visual inspection. Therefore a second component was removed.

ERP segments were created starting 100 ms before and ending 500 ms after saccade onset. A minimum of five trials of either type (correct or error) and in either direction (left or right) was required for inclusion in the statistical analysis. Baseline correction was performed based on the average signal in the 100 ms preceding saccade onset. Segments entailing maximum amplitudes exceeding an absolute value of 100 µV or a voltage step of 50 µV were excluded by means of automatic artefact detection.

In accordance with a previous study [7], ERN amplitudes were derived from the average individual difference waveforms (ERPs on error trials minus those on correct trials). The ERN was defined as the most negative difference wave peak within 160 ms after saccade onset at electrode position FCz.

### Statistical analysis

In a first analysis step, behavioural and ERP data were compared between the groups of patients and controls using *t* tests or Mann–Whitney *U* tests, where appropriate. To better characterize individual patients' impairment patterns and to take into account the small sample size and large age range of the patients, subsequent analysis steps focused on comparing the performance of each patient to an individual age-matched subgroup of controls. If the assumption of normally distributed data in the respective control group was not violated, performance of the single patient was compared to control subjects' performance with a modified *t* test specifically developed for experimental single-case studies [49]. This test controls the Type I error rate for small control groups, as shown by Monte-Carlo simulations [50]. For all analyses, the level of significance was set to *p*<.05 (one-sided).

## Results

### Group level ERP analysis

**Figure 4:**
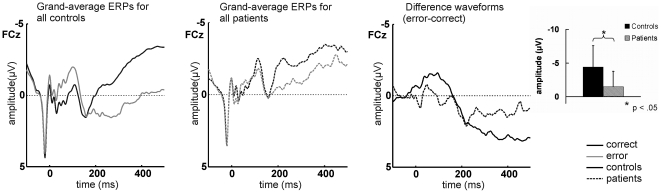
Saccade-locked ERP and difference waveforms for all patients and controls. Saccade-locked grand-average ERP waveforms elicited by correct and erroneous saccades at electrode FCz for the control and the patient group and difference waveforms (error minus correct) at FCz for all patients and controls. Bar charts provide mean ERN amplitudes (error bars represent SDs) which differed significantly between groups, indicating ERN attenuation in patients.

**Figure 5:**
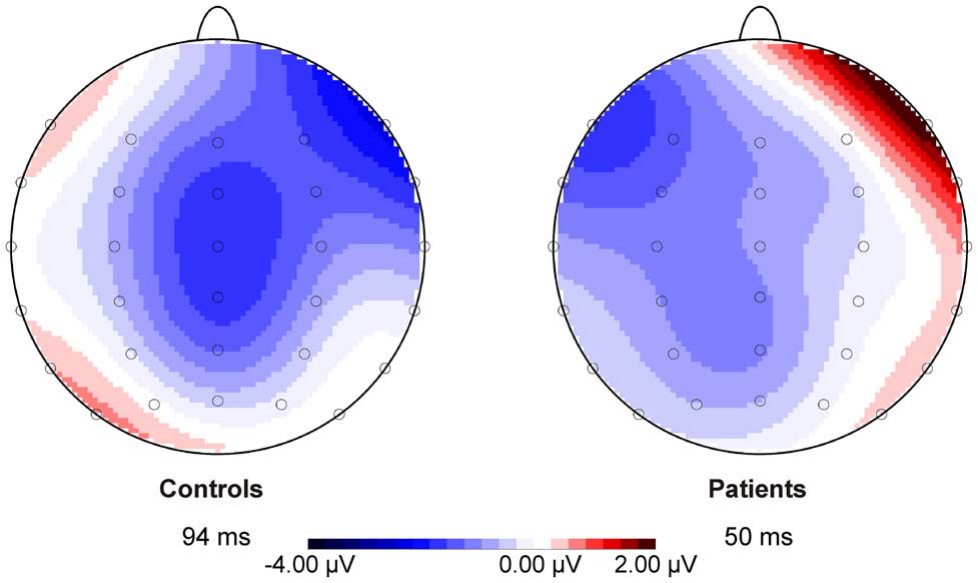
ERN scalp topographies for patients and controls as groups. Topographical maps and latency of the mean most negative peak in the difference waveforms within 160 ms after saccade onset for patients and controls on the group level.

Saccade-locked grand-average ERPs for correct and error trials and the difference waveforms (error minus correct) for the patient and control group are depicted in Figure 4. One-sided *t* tests on mean ERN amplitude (defined as the most negative peak in the difference signal within 160 ms after the saccade) yielded a significant group difference (*t* = -2.139, *p* = .020), indicating reduced ERN amplitudes in the patient group. Topographical maps of the ERN for patients and controls are provided in Figure 5.

### Analysis of behavioural data at group level

Mean SRT of correct trials, percentage and SRT of errors, percentages of corrected and aware errors and correction time for patients and controls are presented in Table 2. A significant group difference for the percentage of errors (*t* = −1.855, *p* = .037) emerged, indicating more errors in the patient group. Error awareness was significantly lower in patients (*U* = 38.00, *p* = .038). Furthermore, there was a significant difference for SRTs on error trials (*t* = −2.715, *p* = .005) and a corresponding trend for SRTs on correct trials (*t* = −2.446, *p* = .079), implying longer saccade latencies in patients. None of the other measures differed significantly between patients and controls on the group level (all *p*>.170).

**Table 2:** Overview of the patients' and controls' mean percentage of correct trials, errors, corrected errors and mean saccadic reaction time (SRT) for correct, erroneous and corrective saccades.

	Correct	Errors	Aware Errors	Errors	Corrected errors	Correction time
	SRT (ms)	%	%	SRT (ms)	%	(ms)
**Controls mean (SD)**	389 (51)	16.5 (11.2)	22.4 (24.4)	246 (41)	81.2 (19.0)	181 (53)
**Patients mean (SD)**	455 (96)^+^	27.2 (19.7)*	7.7 (13.7)*	301 (37)**	76.2 (19.7)	204 (62)
**Patient 1**	375	11.4	4.4	260	95.6	134
*Controls (N = 10)*	377 (55)	17.5 (13.8)	29.4 (29.9)	233 (31)	79.8 (18.34)	185 (44)
**Patient 2**	376	34.6^+^	1.6	242	73.8^+^	173
*Controls (N = 10)*	378 (49)	15.1 (12.1)	25.8 (21.4)	242 (31)	88.4 (7.7)	167 (16)
**Patient 3**	391	27.9	2.4	258	97.6	148
*Controls (N = 10)*	394 (37)	14.5 (10.2)	21.4 (23.4)	255 (37)	84.5 (16.8)	166 (34)
**Patient 4**	581**	10.1	1.7	388*	83.1	260
*Controls (N = 10)*	401 (44)	17.2 (8.2)	12.4 (21.6)	256 (52)	76.3 (24.4)	170 (73)
**Patient 5**	568**	17.0	35.4	374*	50.0	233
*Controls (N = 10)*	403 (46)	16.6 (9.1)	12.5 (21.6)	258 (54)	77.4 (25.3)	170 (73)
**Patient 6**	437	62.3***	0.4	284	57.1	281
*Controls (N = 10)*	402 (45)	19.1 (8.5)	13.4 (21.5)	250 (49)	76.1 (24.9)	173 (77)

Standard deviations (SD) in brackets. *T* tests were performed one-sided. Percentages of aware and corrected errors are provided relative to the overall number of errors.

***p<.0001 ** p<.01 *p<.05, ^+^p<.10.

In order to analyze post-error slowing, the SRT difference between correct trials after correct and erroneous saccades (correct after error – correct after correct) was compared between patients and controls. Positive values indicate slower responding following errors, i.e. post-error slowing (see Table 3 for means and SDs of SRTs). There was a trend towards significantly enhanced post-error slowing in the patients relative to the controls (*t* = −1.385, *p* = .088).

**Table 3:** Post-error slowing data for patients and controls: Mean saccadic reaction times (SRTs) in succeeding correct trials following errors and correct saccades and the mean difference in SRTs.

	Post-error slowing: SRTs (ms)		SRT difference
	correct after correct	correct after error	
**Controls mean (SD)**	389 (49)	388 (62)	−1.3 (35.0)
**Patients mean (SD)**	442 (106)	464 (124)	21.9 (47.7)^+^
**Patient 1**	376	365	−11.0
*Controls (N = 10)*	379 (54)	373 (52)	−6.2 (17.2)
**Patient 2**	366	338	−27.6^+^
*Controls (N = 10)*	378 (49)	385 (59)	7.6 (21.6)
**Patient 3**	392	389	−3.3
*Controls (N = 10)*	392 (37)	406 (47)	13.8 (17.8)
**Patient 4**	576	627	50.6
*Controls (N = 10)*	402 (40)	392 (74)	−9.5 (52.0)
**Patient 5**	580	600	21.4
*Controls (N = 10)*	403 (41)	396 (76)	−6.8 (52.1)
**Patient 6**	360	467	101.8*
*Controls (N = 10)*	401 (41)	397 (76)	−4.4 (52.6)

Standard deviations (SD) are presented in brackets. *T* tests were performed one-sided.

*p<.05, ^+^ *p*<.10.

### Single-case ERP analyses

**Figure 6:**
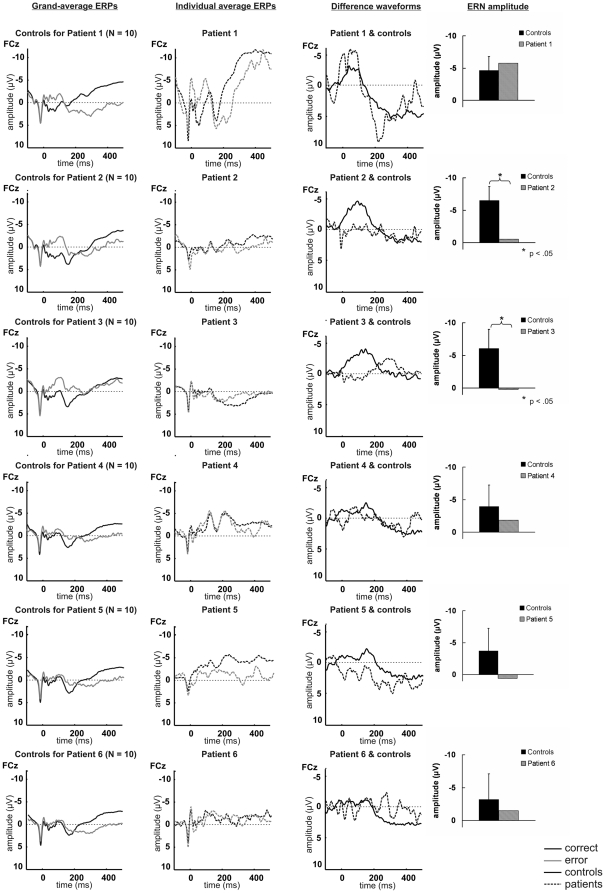
Saccade-locked ERP and difference waveforms for individual patients and respective control groups. Saccade-locked average and grand-average ERP waveforms elicited by correct and erroneous saccades at electrode FCz for individual patients and their respective control groups, and difference waveforms (error minus correct) at FCz for individual patients and corresponding controls. Bar charts provide mean ERN amplitudes for individual patients and respective controls. Analyses revealed reduced ERN amplitudes in Patient 2 and Patient 3 compared to corresponding samples of controls.

**Figure 7:**
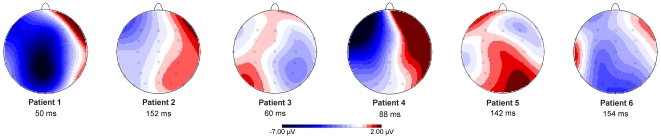
ERN scalp topographies for individual patients. Topographical maps and latency of the mean most negative peak in the difference waveforms within 160 ms after saccade onset for individual patients.

Figure 6 shows saccade-locked average ERPs on correct and error trials for individual patients, grand averages for the respective control groups and the corresponding difference waveforms. Single-case *t* tests showed significantly lower ERN amplitudes in Patient 2 (*t* = 2.639, *p* = .013) and Patient 3 (*t* = 2.027, *p* = .037) compared to their respective control groups. None of the other patients differed significantly from his/her corresponding controls (all *p*>.137). ERN scalp topographies for individual patients are depicted in Figure 7.

### Single-case analyses of behavioural data

For Patient 2, trends for a significantly higher error rate (*t* = 1.547, *p* = .078) and for significantly fewer corrected errors (*t* = −1.801, *p* = .052) emerged in comparison to the respective control group. Patients 4 and 5 both showed significantly longer SRTs on correct and error trials (Patient 4 - correct: *t* = 3.857, *p* = .002; error: t = 2.409, *p* = .004; Patient 5 - correct: *t* = 2.077, *p* = .002; error: t = 2.409, *p* = .034). Finally, the error rate was significantly increased for Patient 6 (*t* = 4.857, *p*<.0001). None of the other comparisons between single patients and the respective control groups revealed significant or near-significant differences with respect to saccade latencies and error rates (all *p*>.104).

Because error awareness data were not normally distributed for the individually matched control groups, the single case *t* tests could not be applied to examine the performance of individual patients. However, on the descriptive level, error awareness was very low (<5%) in all patients except for Patient 5. Error awareness was particularly low in Patient 2, whose score was lower than the lowest score in the respective control group.

With respect to post-error slowing the *t* tests showed a significantly stronger effect for Patient 6 compared to the respective sample of control subjects (*t* = 1.922, *p* = .043). Furthermore, there was a trend for reduced post-error slowing in Patient 2 (*t* = −1.560, *p* = .077). None of the other patients differed significantly from the respective controls with regard to post-error slowing (all *p*>.150).

### Direction-specific analyses in individual patients

To elucidate whether the significant ERN amplitude reductions observed in two individual patients, i.e. Patients 2 and 3, were dependent on saccade direction, separate exploratory analyses were conducted for trials with left- and rightward pro- and antisaccades.

For Patient 2, ERN amplitudes were significantly attenuated on rightward trials compared to the group of control subjects (Patient 2: −1.39 µV, controls: mean = −6.89 µV, SD = 2.83; *t* = 1.853, *p* = .048), and a corresponding trend emerged for leftward trials as well (Patient 2: −0.67 µV, controls: mean = −7.36 µV, SD = 3.77; *t* = 1.692, *p* = .062). The error rate was significantly increased for rightward trials (Patient 2: 19.2, controls: mean = 7.3 (SD = 6.1); *t* = 1.862, *p* = .048). No significant difference was observed for leftward trials (Patient 3: 15.4, controls: mean = 7.7 (SD = 6.5; *p* = .143). The rates of corrected errors did not differ significantly from the respective controls for trials in either direction (both *p*>.228).

For Patient 3, tests yielded significantly reduced ERN amplitudes on both leftward (Patient 3: −1.38 µV, controls: mean = −7.81 µV, SD = 2.94; *t* = 2.085, *p* = .033) and rightward trials (Patient 3: 0.17 µV, controls: mean = −7.18 µV, SD = 3.70; *t* = 1.894, *p* = .045). The error rate was significantly increased for rightward trials (Patient 3: 17.1, controls: mean = 7.1 (SD = 5.1); *t* = 1.870, *p* = .047). For leftward saccades, no significant difference was found (Patient 3: 10.8, controls: mean = 7.4 (SD = 5.9); *p* = .298), and the rates of corrected errors did not differ between Patient 3 and the respective controls in either direction (both *p*>.136).

### Alertness and working memory

Table 4 shows mean alertness and verbal as well as visual short-term and working memory scores on the group level and for individual patients and their respective subgroups of controls. On the group level, patients and controls did not differ significantly in regard to measures of short-term or working memory (all *p*>.122). There was a significant group difference for phasic alertness (*t* = −2.169, *p* = .038) and a trend towards a significant difference for tonic alertness (*t* = −2.009, *p* = .053), both indicating higher reaction times for patients.

**Table 4:** Overview of the patients' and controls' mean performance on the alertness, short-term and working memory tasks.

	Alertness (reaction time)		Visual memory		Verbal memory	
	tonic (ms)	phasic (ms)	short-term	working	short-term	working
**Controls mean (SD)**	304 (52)	295 (54)	9.1 (2.3)	8.4 (2.1)	8.9 (2.0)	7.6 (1.8)
**Patients mean (SD)**	349 (55)^+^	345 (54)*	8.0 (1.4)	7.0 (2.0)	8.0 (2.1)	6.7 (1.6)
**Patient 1**	319	305	10	9	9	9
*Controls (N = 10)*	285 (36)	282 (50)	9.6 (2.2)	8.7 (2.4)	8.4 (1.4)	7.4 (1.7)
**Patient 2**	298	279	11	9	10	10
*Controls (N = 10)*	307 (55)	292 (55)	8.2 (2.7)	8.1 (1.6)	9.1 (1.8)	7.9 (1.8)
**Patient 3**	285	307	9	6*	9	5
*Controls (N = 10)*	315 (63)	309 (6)	8.7 (2.5)	8.6 (1.0)	8.9 (1.7)	7.0 (1.9)
**Patient 4**	324	322	7	6	7	6
*Controls (N = 10)*	315 (61)	310 (64)	9.2 (1.7)	8.0 (2.2)	9.0 (2.7)	7.2 (2.2)
**Patient 5**	384	419^+^	5*	6	8	5
*Controls (N = 10)*	317 (59)	314 (61)	9.1 (1.5)	7.9 (2.1)	8.7 (2.6)	7.0 (1.9)
**Patient 6**	412^+^	397^+^	7	5	6	8
*Controls (N = 10)*	313 (56)	301 (53)	8.6 (1.7)	7.4 (2.1)	8.3 (2.5)	6.8 (1.9)

Standard deviations (SD) provided in brackets.

*p<.05, ^+^ *p*<.10.

Single case *t* tests did not yield significant differences between individual patients and respective control groups for measures of verbal short-term or working memory (all *p*>.283). Compared to the respective control samples, there were trends for significantly increased RTs for both tonic (*t* = 1.700, *p* = .060) and phasic alertness (*t* = 1.731, *p* = .059) in Patient 6, and for phasic alertness only in Patient 5 (*t* = 1.639, *p* = .068). Patient 3 scored significantly lower on the visual working memory task (*t* = −2.417, *p* = .039), and Patient 5 scored significantly lower on the visual short-term memory task (*t* = -2.572, *p* = .030). None of the other comparisons yielded any significant differences (all p>.122).

## Discussion

The present study investigated error processing on an antisaccade task in patients with focal ischemic thalamic lesions and healthy control participants. ERPs in response to correct anti- and erroneous prosaccades were analyzed. Based on previous findings indicating a critical role of the thalamus for online monitoring of saccadic eye movements, it was hypothesized that the ERPs in the patients would distinguish less reliably between errors and correct performance than the ERPs of control subjects.

In line with the hypothesis, ERN amplitudes were found to be reduced in the patients relative to controls. On the behavioural level, error rates were significantly higher and error awareness was reduced in the patients. Neither ERN nor behavioural data yielded clear evidence of direction specific effects in the four patients with unilateral lesions.

Higher error rates might reflect increased uncertainty about the response to be made. In accordance with the error detection account, ERN attenuation has previously been linked to response uncertainty, with reduced ERN and enhanced CRN amplitudes when the correctness of a response could not be verified due to limited available information [22]. Error monitoring was less reliable when perceptual discrimination of two stimuli was more difficult or when attentional resources were strained by dual task demands [22]. However, ERN attenuation in the current study cannot be explained in terms of such a response uncertainty, as the vast majority of erroneous prosaccades in both controls and patients was immediately followed by a corrective saccade, a clear indication that the participants' representations of the correct response in a given context was generally intact.

It may be argued that the performance of corrective saccades constitutes a source of confound in the comparison of error and correct trials, as the mean correction time was quite short (ca. 180 to 200 ms, see Table 2) so that a proportion of these saccades was started in the ERN analysis time window (<160 ms after saccade onset). In previous studies examining error processing by means of an antisaccade task, potential effects of corrective saccades were not discussed [7], [8], although similar mean correction times were reported and similar time windows for ERN analysis were applied. Importantly, patients and controls did not differ with regard to percentage or latency of corrective saccades in the present study, arguing against an impact of corrective saccades on the ERN result pattern in the between-group comparison. Nevertheless, additional analyses were conducted to exclude a potential confound. We repeated the ERN analysis including only those trials with no corrective saccade or with correction times larger than 180 ms. Overall, the mean percentage of trials not fulfilling the selection criteria was comparable in patients and controls (patients: mean = 47.4, SD = 21.2; controls: mean = 48.6, SD = 20.6). One control subject and one patient (Patient 1) had to be excluded from this analysis because more than 80% of error trials had to be discarded. Notably, Patient 1 did not show evidence of ERN reduction. Additionally, Patient 1 showed a very short ERN latency, so that the cut-off criterion of 180 ms was too strict in this patient. For the remaining five patients and 27 controls the analysis corroborated the result of reduced ERN amplitudes in the patients found with the original data set including all trials.

Furthermore, the increased error rate in the patients itself may also have affected ERN amplitude. Previous research has yielded inconsistent results in regard to the relationship between error rates and ERN amplitude. While Hajcak et al. [51] found an inverse relationship, i.e. a reduction in ERN amplitude with increasing error rates, Pailing et al. [52] failed to observe such a pattern. Instead, a positive correlation between impulsivity as indicated by smaller reaction time differences between correct responses and errors and ERN amplitude was postulated, linking the ERN to response control and a remedial action system [52]. Exploratory analysis of the relationship between the error rate and ERN amplitude in the present sample (patients and controls pooled) did not yield a significant correlation (*r* = .228, *p* = .195). Moreover, only one of the two patients (Patient 2) for whom ERN amplitude in the difference waveform was significantly reduced relative to the respective control sample showed significantly increased overall error rates. Taken together, these observations appear to suggest that altered error processing in thalamic lesion patients as indicated by reduced ERN amplitudes and increased error rates on the antisaccade task, may constitute – at least partially – independent deficits.

### The role of thalamo-cortical connections for efference copy processing

Fast error processing as indicated by the ERN requires exact information about the movement which has just been executed or is about to be executed. As was outlined in the introduction, efference copies are likely to provide such information, in particular because the ERN has a very short latency and can therefore not rely on proprioceptive or external feedback, which would take much longer to be processed. Pathways conveying efference copy information have only rarely been studied to date. However, in the monkey brain one pathway carrying efference copy signals associated with saccadic eye movements from the SC to the FEF with a relay in the lateral thalamic MD nucleus has been identified [30]–[32]. Monkeys with MD lesions showed a deficit in a saccadic double-step task requiring the use of efference copy information for the programming of two successive saccades. In human subjects, thalamic lesions have been shown to impair performance in this task as well [34], [36]. The most pronounced deficit was, however, observed in patients with lesions to the VL region [34]. It has been proposed that the SC-FEF pathway might pass through more ventrolaterally located nuclei rather than MD in humans [53], but this hypothesis still needs to be corroborated. This may include VL as well as the centrolateral nucleus (CL), which is located exactly between the lateral MD and the ventrolateral nucleus. CL is part of the intralaminar group (ILN) and receives input from the cerebellum and BG, and it projects to the frontal and parietal lobe [54], [55] (see [56] for a review). The ILN has been suggested to be directly involved in saccade processing ([56] for a review). In the present study sample, thalamic lesions may have disrupted connections of SC, cerebellum and basal ganglia to the frontal and parietal cortex, leading to a less accurate representation of the actually executed response and ultimately to less distinct ERPs for correct prosaccades and erroneous antisaccades, as manifested in reduced ERN (i.e. difference wave) amplitudes.

A very recent finding of reduced ERN amplitudes in thalamic lesion patients in a flanker task requiring fast hand responses (button presses) shows that the role of the thalamus in performance monitoring is not restricted to saccades [57]. Nevertheless, it is conceivable that similar mechanisms apply. Also for hand movements, efference copy information may pass through the thalamus. In this study, most pronounced ERN reductions were observed after lesions to the ventral anterior and ventral lateral anterior nuclei.

Analyses of ERPs in individual patients of the present study did, however, not yield a clear pattern with respect to the thalamic nuclei particularly involved in saccade monitoring. The most pronounced ERN reduction was found in Patients 2 and 3. Patient 3 suffered from damage to VL primarily on the left and less extensively also on the right side. In Patient 2, the right MD nucleus appeared to be primarily affected, which is consistent with deficits in using efference copy information previously reported for a single MD lesion patient [34]. Detailed lesion analysis revealed, however, that VL was also affected in Patient 2. Overall, the result patterns of individual patients thus may suggest a more important role of the ventrolateral thalamus, because significant ERN reduction is only observed in patients, in whom VL is affected (with the exception of Patient 4), while patients with exclusive MD involvement (Patients 1, 5 and 6) did not show an ERN reduction. However, in the older patients 4, 5 and 6, potential ERN reduction may be masked by age effects, which are also present in the control group (see below), so that firm conclusions on the differential contributions of specific thalamic nuclei cannot be drawn based on the present findings.

The pattern of results is also reflected in the corresponding topographical maps, both on the group level and in individual patients (see Figures 5 and 7). In Patients 2, 3 and 5, the maps reflect the complete absence of a (relative) negativity for error trials. In Patient 1, who showed an intact ERN, a clear central negativity is observed. In Patients 4 and 6, in whom a (not significantly) reduced, but clearly visible ERN was found, the topography was altered, with a more prominent negativity over the left scalp (most pronounced in Patient 4). As already mentioned above, lesions to the thalamus and the VL in particular are likely to affect pathways possibly relaying efference copy information other than the SC – FEF pathway. One alternative pathway may involve cerebello-thalamo-cortical connections linking oculomotor regions of the cerebellar dentate nucleus to the FEF [33], [34], PFC and premotor cortex [58]. The cerebellum is a likely source of efference copy information about ongoing saccades. Cerebellar neurons code the amplitude of the actually performed (rather than the planned) saccade [59], and repetitive transcranial magnetic stimulation of the posterior cerebellum [60] as well as cerebellar lesions disrupt saccadic adaptation [61], [62]. The ventrolateral thalamus is the main target of cerebellar output projections and hence considered as ‘motor thalamus’ [63], but dense cerebello-intralaminar connections have also been reported [64] (for a review see [56]). Furthermore the cerebellum has recently been suggested to mediate error and post-error processing in ventrolateral prefrontal cortex through functional connections with the supplementary motor area and the thalamus [65], emphasizing the role of cerebello-thalamo-cortical connections for performance monitoring.

### Error awareness and the ERN

The ERN has been shown to be independent of error awareness, i.e. subjects do not need to be aware of having made an error [7], [8], and they can be aware of errors and yet not produce an ERN [66]. Nevertheless, the process reflected by the ERN may be a prerequisite for conscious error detection. In the present study, error awareness was reduced in the patients, providing further evidence for altered error processing. This finding is in line with a recent study reporting reduced conscious error detection on a flanker task in thalamic lesion patients [57]. It has to be noted that in the present study error awareness was assessed in an ‘all or nothing’ fashion by asking participants to only press a button if they had sensed an erroneous prosaccade. This procedure does not allow assessment of varying degrees of uncertainty about the performed saccade and largely relies on the subject's individual proneness to respond to a mere feeling of having made an error.

Although efference copies may contribute to error awareness, error detection in the antisaccade task as applied here did not necessarily have to rely on efference copy signals. The square frames indicating target and cue locations remained visible throughout the cue-target interval and could be used as landmarks in error detection. Efference copy information, as it is readily available once a saccade is initiated, is likely to contribute to fast processing of saccade-related information, while other sources of information are certainly also exploited when it comes to error correction and conscious error detection.

### Thalamo-prefrontal connections and executive control

Disturbed error monitoring due to a disruption of connections between the thalamus and the prefrontal cortex (PFC) may provide an alternative explanation for the ERN reduction observed in the present study. Dense reciprocal connections between MD and PFC suggest that MD may also contribute to executive aspects of behaviour [58]. Interestingly, lesions of the lateral PFC have been shown to be associated with less distinct correct-trial and error-trial ERPs [67]. Along similar lines, Seifert and colleagues [57] could show that ERN reductions in a flanker task in thalamic lesion patients were most pronounced when thalamic damage affected a region with dense connections to the anterior midcingulate cortex. The authors stated that the thalamus relays motor action related information from the striatum and the cerebellum to the cingulate cortex. This input may inform the cingulate cortex about ongoing movements and enable a comparison between desired and actually performed responses, possibly also recruiting action representations in the PFC. Such an interpretation is well compatible with the assumption that the thalamus relays efference copies of motor commands, on which the present study is based.

As was already outlined above, deficits in the inhibition of reflexive prosaccades, as observed in the patients of the present study, may be independent of ERN reduction. Kunimatsu and Tanaka [68] recently showed that the ventroanterior (VA) and VL thalamic nuclei are critically involved in antisaccade performance in monkeys [68]. Neuronal activity in VL and VA was strongly enhanced for anti- compared to prosaccades, and inactivation of these nuclei led to increased error rates, whereas inactivation of neurons in MD did not alter antisaccade performance [68]. As Seifert et al. [57] did not show increased error rates in a flanker task in thalamic lesion patients, the role of the thalamus in response inhibition may be specific for saccades. On the other hand, Condy et al. [69] failed to find increased rates of reflexive prosaccades for thalamic lesion patients, apparently contradicting the findings of the present study.

With respect to the thalamic nuclei associated with increased error rates, no clear pattern emerged in the present sample of patients. However, in contrast to the findings in monkeys, VL does not appear to have a prominent role in this respect. Patient 3, in whom the lesion involved VL bilaterally, did not show a significantly increased error rate, whereas Patients 2 and 6, whose lesions affected primarily MD, showed markedly enhanced rates of erroneous prosaccades, with the error rate reaching 62% in Patient 6. This exceptionally high error rate may be suggestive of a deficit in inhibitory control in Patient 6. The PFC has long been known for its importance for executive control and is therefore likely to be involved in the suppression of reflexive prosaccades. Indeed, patients with lesions to the dorsolateral PFC showed increased error rates on an antisaccade task, while patients with lesions to posterior parietal cortex, supplementary motor area or FEF did not [70]. It has been hypothesized that the dorsolateral PFC may exert inhibitory control on the saccade-generating SC through prefronto-tectal pathways involving the internal capsule [69].

### Relation between age and ERN amplitude

A common finding in ERN research is an attenuation of the component with increasing age [71]–[73]. As depicted in Figure 6, the present data support this effect, with smaller ERN amplitudes in older (e.g. the control groups for Patient 4, Patient 5 and Patient 6) compared to younger participants (e.g. the control groups for Patient 1, Patient 2 and Patient 3). In older patients, effects of increased age and thalamic damage may therefore have accumulated. ERN reductions in healthy controls provided less ‘room’ for substantial ERN attenuation due to thalamic damage. Such a ‘floor effect’ may have masked potentially significant ERN alterations in Patients 4, 5 and 6, somewhat weakening the present findings for individual patients.

On the descriptive level, however, neither of the older patients appeared to exhibit a clear ERN in the difference waveforms. Thus, although the ERN effects were strongest in Patients 2 and 3, low ERN amplitudes are a general finding in the patient group, and reduced ERN amplitudes in the older patients (Patients 4, 5 and 6) contributed to the significant difference between patients and controls on the group level (see Figure 6).

### Saccadic reaction time and post-error slowing

Finally, SRTs were generally longer in the patients and a trend for more pronounced post-error slowing was found. The former finding was probably related to general response slowing in the patients, as indicated by increased RTs in the alertness task. On the individual level, SRTs were longer only for Patient 4 and Patient 5 compared to the respective control samples. However, such an individual increase in SRTs was not associated with error rate changes, as both patients did not differ from their respective controls in this respect.

The latter finding was surprising, but single-case analyses suggested that the effect was mainly driven by a single patient – Patient 6 – in whom post-error slowing was particularly strong. Control participants, on the other hand, did not show a pronounced post-error slowing effect. While this observation seems to contradict earlier findings [7], [8], the lack of post-error slowing is likely due to very low error awareness in the present sample. Indeed, previous studies have reported a pronounced post-error slowing effect only for aware as compared to unaware errors [7], [8].

### Conclusion

To conclude, the present data provide evidence for altered error processing on an antisaccade task in patients with focal thalamic lesions. Altered error processing primarily refers to smaller differences between ERPs of erroneous prosaccades and correct antisaccades. Deficit patterns in individual patients tentatively suggest an important role of the ventrolateral thalamus in online saccade monitoring. Because of the small sample size, age effects on the ERN and interindividual variability with regard to lesion size and location no firm conclusions can be drawn in this respect and the exact functional contributions of different thalamic substructures remain to be more fully determined. The present data further support the notion that the ERN is based on efference copy signals, which may be compromised due to the thalamic lesion. Recent findings on error processing in thalamic lesion patients in the context of a different task further suggest that the thalamic function as efference copy relay may not be restricted to saccades [57]. Together with the present findings, it can be concluded that the thalamus plays a critical role in performance monitoring.
